# Direct Wafer Bonding and Its Application to Waveguide Optical Isolators

**DOI:** 10.3390/ma5050985

**Published:** 2012-05-24

**Authors:** Tetsuya Mizumoto, Yuya Shoji, Ryohei Takei

**Affiliations:** Department of Electrical and Electronic Engineering, Tokyo Institute of Technology, 2-12-1 Ookayama, Meguro-ku, Tokyo, 152-8552, Japan; E-Mails: shoji.y.ad@m.titech.ac.jp (Y.S.); r.takei@aist.go.jp (R.T.)

**Keywords:** waveguide device, magneto-optics, direct wafer bonding, optical isolator

## Abstract

This paper reviews the direct bonding technique focusing on the waveguide optical isolator application. A surface activated direct bonding technique is a powerful tool to realize a tight contact between dissimilar materials. This technique has the potential advantage that dissimilar materials are bonded at low temperature, which enables one to avoid the issue associated with the difference in thermal expansion. Using this technique, a magneto-optic garnet is successfully bonded on silicon, III-V compound semiconductors and LiNbO_3_. As an application of this technique, waveguide optical isolators are investigated including an interferometric waveguide optical isolator and a semileaky waveguide optical isolator. The interferometric waveguide optical isolator that uses nonreciprocal phase shift is applicable to a variety of waveguide platforms. The low refractive index of buried oxide layer in a silicon-on-insulator (SOI) waveguide enhances the magneto-optic phase shift, which contributes to the size reduction of the isolator. A semileaky waveguide optical isolator has the advantage of large fabrication-tolerance as well as a wide operation wavelength range.

## 1. Introduction

An optical isolator allows light waves to propagate only in a specified direction. It plays an essential role in optical circuits by preventing light waves from propagating in the undesired direction. For instance, the optical isolator protects laser diodes from reflected light and suppresses the injection noise of a laser that is caused by launching a reflected emission into the laser cavity [1]. Currently, commercially available optical isolators employ the rotation of polarization, where the essential function of nonreciprocity is provided by the magneto-optic Faraday effect. They are composed of bulk optics, and, hence, are not suitable for integrating with other optical devices. The optical isolator must be realized in a waveguide form for integration. 

Restrictions related to materials should first be taken into account for the integrating optical devices. When devices are composed of a single material system, for instance III-V compound semiconductors, optically active as well as passive devices can be monolithically integrated as illustrated in [2]. A magneto-optic effect is important for obtaining the nonreciprocal function of an optical isolator. Magneto-optic garnet crystals are commonly used to obtain the optical nonreciprocal function in optical fiber communication wavelength ranges, since they have a large magneto-optic effect and low optical absorption [3]. However, it is hard to grow a single-crystalline magneto-optic garnet on commonly used optical waveguide platforms made of III-V compound semiconductors, silicon and silica. One of critical issues for integrating the optical isolator is to integrate a magneto-optic garnet on the waveguide platforms. In order to resolve this issue, several approaches are investigated, which include deposition and bonding techniques. 

Another issue to be considered for integrating optical isolators is the compatibility of the device structure with the platform of other optical devices. That is, most optical devices consist of III-V compound semiconductor, silicon or silica guiding layers. The waveguide alignment between an optical isolator and other devices can be achieved by a lithography process, when the optical isolator is fabricated on these waveguide platforms. A nonreciprocal loss optical isolator, which is composed of a ferromagnetic cladding layer together with a built-in semiconductor optical amplifier, exhibits this excellent compatibility with an optically active device composed of III-V compound semiconductors [4,5,6,7,8,9,10,11]. The nonreciprocal loss isolator requires optical amplification to cancel the large optical absorption of ferromagnetic material. The optical amplification is obtainable by the current injection in case of III-V compound semiconductors. However, this is not the case for other waveguide platforms such as a silicon-based waveguide. Since the optical amplification is not easily obtained in the SOI (silicon-on-insulator) waveguide, the loss-compensation configuration is not directly applicable to this waveguide platform. Because of this, the applicability of the nonreciprocal loss optical isolator is somewhat limited.

The large tolerance for fabrication errors is an important issue from a practical viewpoint. A semileaky waveguide isolator has the great advantage of a fairly large fabrication tolerance together with a wide operation wavelength range [12]. It is necessary to use a magneto-optic material and a birefringent crystal stacked onto each other in a waveguide. A direct bonding technique enables the creation of this isolator.

In Section 2, the present paper reviews approaches to integratable optical isolators. Then, the surface activated direct bonding technique is described as a powerful tool to integrate a magneto-optic garnet on dissimilar crystals. The conditions developed for bonding a magneto-optic garnet on silicon and III-V compound semiconductors are described in Section 3. In Section 4, an interferometric waveguide optical isolator is described focusing on a silicon waveguide platform. In Section 5, the semileaky waveguide optical isolator is described as a fabrication tolerant waveguide optical isolator. 

## 2. Approaches to Integratable Optical Isolators

Waveguide optical isolators have been studied for realizing integratable optical isolators. In 1972, Wang *et al*. reported the theoretical study of mode conversions in magneto-optic waveguides [13]. Warner reported the design of waveguide optical isolators based on a TE-TM mode-conversion in 1973 [14]. In 1975, Hepner *et al*. measured the TE-TM mode conversions in a magneto-optic garnet waveguide induced by external magnetic fields applied in the longitudinal (Faraday) and transverse (Cotton-Mouton) directions [15]. In 1977, Castera *et al*. demonstrated a waveguide optical isolator using the Faraday and Cotton-Mouton effects in a gadolinium-gallium substituted yttrium iron garnet (YIG) [16]. 

In order to obtain a TE-TM mode conversion sufficient for optical isolator operation in these devices, the phase matching between the modes concerned is important [17]. Wolfe *et al*. proposed a *quasi* phase matching technique in order to overcome the phase mismatch between TE and TM modes by reversing the magnetization of garnet periodically along the light propagation direction [18]. Also, Wolfe *et al*. demonstrated a magneto-optic waveguide of zero liner birefringence by controlling the shape birefringence in a multiple-layer bismuth-substituted YIG (Bi:YIG) waveguide [19]. In 1988, Ando *et al*. fabricated the magneto-optic garnet waveguide in which the Faraday and Cotton-Mouton sections are realized, by making use of the localized control of magnetization direction, for nonreciprocal and reciprocal TE-TM mode conversions, respectively [20]. The phase matching between TE and TM modes was achieved by balancing the linear birefringence of waveguide with the stress and growth induced birefringence of the garnet layer. They demonstrated an isolation of 12.5 dB at a wavelength of 1.15 μm. 

All these investigations were done in waveguides composed of magneto-optic garnet guiding layers. The garnet-based waveguide optical isolator can be integrated into other waveguide platforms, if a hybrid integration technique is applied as shown in [21]. In this approach, the alignment between devices could be a serious problem, since a typical accuracy of a sub-micron is required for low coupling losses. If the waveguide of the optical isolator is fabricated in the same lithography process simultaneously with other devices, the alignment issue can be avoided. The fabrication of an optical isolator is accomplished by integrating a magneto-optic garnet into the waveguide after completing the waveguide fabrication. Deposition and bonding techniques are investigated to integrate a magneto-optic garnet on commonly used waveguide platforms. 

The epitaxial growth of a magneto-optic garnet on silicon and III-V compound semiconductors is a quite challenging work, since there exist large mismatches in physical properties between the garnet and these semiconductors. A magneto-optic garnet has not been grown on semiconductors with good crystallinity, sufficiently large magneto-optic effect and practically low optical absorption [22,23,24]. Bi *et al*. succeeded in depositing a magneto-optic garnet bilayer on silicon by using a two-step deposition method. The deposited layer was composed of a polycrystalline cerium-substituted YIG (Ce:YIG) layer with a thin YIG buffer layer. The Ce:YIG layer exhibited a rather large optical attenuation and the magneto-optic effect reduced to 30% of that found in a single-crystalline Ce:YIG [25,26].

When integrating a magneto-optic garnet into other materials for the optical waveguide application, a uniform contact is needed between materials with sufficient bond strength for obtaining an interaction with the magneto-optic garnet. Several bonding techniques have been investigated in order to achieve this. It should be noted that the bonding technology enables one to bond dissimilar crystals independently of crystallographic orientations.

Ghosh *et al*. intensively studied the adhesive bonding of magneto-optic garnets on silicon waveguides using benzocyclobutene (BCB) as an adhesive material [27]. In case of the adhesive bonding with a typical BCB thickness of 100 nm, the obtainable magneto-optic effect is greatly reduced compared with the case of direct bonding. Also, it is needed to accurately control the thickness of the adhesive layer for obtaining the designed nonreciprocal effect. 

Direct bonding techniques avoid such a problem. Anodic bonding is applied to the bonding of silicon and silica. The anodic bonding is carried out by clamping materials between two electrodes at elevated temperatures. A high DC voltage is applied between the electrodes to create an electrical field in the materials, by which ions are displaced at an elevated temperature from the bonding surface. The displacement of ions makes the surface highly reactive forming a solid chemical bond. In the anodic bonding, the interdiffusion of ions could be a problem, since it changes the properties of materials. 

In fusion bonding, the materials are first forced into intimate contact and then are annealed at high temperature, which results in a solid bond. Since the materials are annealed at high temperature, the difference in the coefficient of thermal expansion results in a large stress in the materials. This is why the fusion bonding is not appropriate for a wafer combination of garnet and III-V compound semiconductors or silicon.

Izuhara *et al*. developed a hydrophilic bonding of Bi:YIG on silicon with a bond strength of ~200 kg/cm^2^ [28]. Roh *et al*. succeeded in bonding Ce:YIG on the InP capping layer of multimode interference coupler using a hydrophilic bonding technique [29]. They demonstrated an isolation of 2.9 dB using magneto-optic nonreciprocal phase shift induced by Ce:YIG. Yokoi *et al*. bonded Ce:YIG on a GaInAsP Mach-Zehnder interferometer (MZI) waveguide using a hydrophilic bonding technique to demonstrate an isolation of 4.9 dB employing a nonreciprocal phase shift in the MZI [30]. A magneto-optic garnet was directly bonded even on a glass substrate, which enabled the integration of optical nonreciprocal devices into glass waveguides [31].

A surface activated direct bonding technique is known to be a powerful tool to realize a tight contact between dissimilar crystals. This technique has the potential advantage that dissimilar materials are bonded at low temperature or without an annealing process [32,33]. Since a garnet has a relatively large thermal expansion coefficient of 9.2 × 10^−6^ K^−1^ (Gd_3_Ga_5_O_12_) compared with InP 4.6 × 10^−6^ K^−1^ and silicon 2.6 × 10^−6^ K^−1^, thermal stress at the bonding interface causes cracking of the specimen and/or debonding from the interface in the heating and cooling processes. Thus, lowering the annealing temperature is of great importance. The surface activated direct bonding technique was developed to integrate Ce:YIG into III-V compound semiconductors, silicon, and LiNbO_3_ [12,34,35].

## 3. Surface Activated Direct Bonding

**Figure 1:**
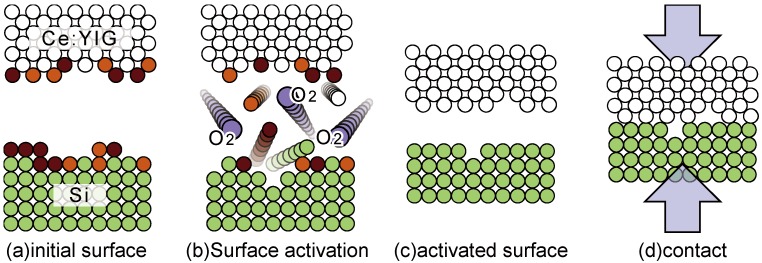
Schematic illustration of surface activated direct bonding process.

The schematic process of the surface activated direct bonding is illustrated in Figure 1. The surfaces of wafers to be bonded are activated in a vacuum chamber. Then, they are brought into contact at room temperature in the chamber. Contacted wafers are pressed at elevated temperatures to obtain a firm bonding. There are several ways of surface activation such as the irradiation of an argon ion beam and argon or nitrogen plasma. It was found that exposing wafers to oxygen plasma was effective for wafer combinations of III-V compound semiconductors and a magneto-optic garnet as well as silicon and a magneto-optic garnet. 

The surface smoothness is an important issue for realizing successful bonding. Takei *et al*. examined the effect of pre-cleaning and surface activation process on the roughness of wafer surfaces [36]. A 0.5-μm-thick Ce:YIG single-crystalline layer was grown on a 450-μm-thick (111)-oriented (GdCa)_3_(GaMgZr)_5_O_12_ (SGGG) substrate by a sputter epitaxy growth technique. They used a 350-μm-thick (100)-oriented InP substrate as a representative of III-V compound semiconductor, a 625-μm-thick (100)-oriented SOI wafer and a 400-μm-thick x-cut LiNbO_3_ substrate in the experiment. The thickness of the silicon layer was 220 nm in the SOI wafer. The details of pre-cleaning are described in [36]. Ce:YIG, InP, SOI and LiNbO_3_ wafers were cut into 20 × 20 mm^2^ pieces.

**Figure 2:**
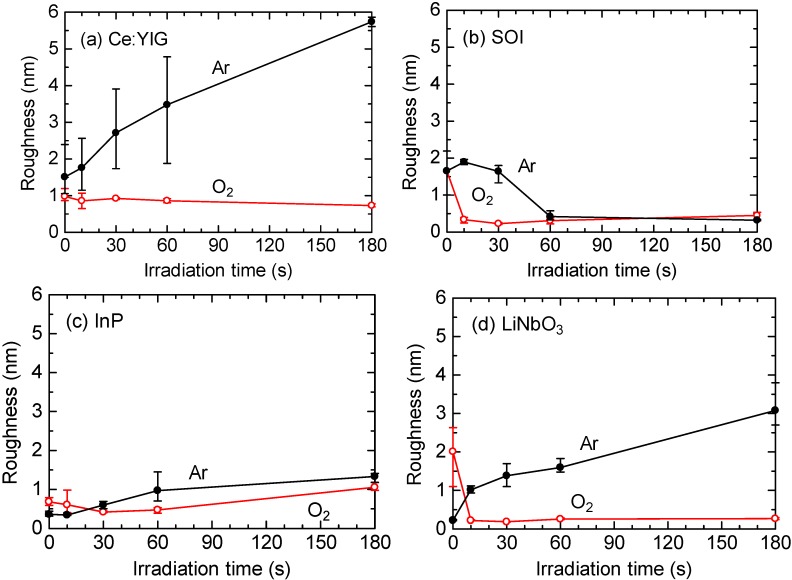
Measured surface roughness of (**a**) Ce:YIG, (**b**) silicon-on-insulator (SOI), (**c**) InP, and (**d**) LiNbO_3_ as a function of plasma irradiation time [36].

Oxygen or argon gas was supplied with a flow rate of 100 sccm into a plasma reactor chamber with a pressure of 120 Pa. In order to generate plasma, a 13.56 MHz RF power of 400 W was applied to an electrode of 4 inches in diameter, on which wafer samples were placed. The roughness of the wafer surfaces was measured by atomic force microscope (AFM). The surface roughness, which is defined by the average of rms values obtained by the AFM measurement, is shown in Figure 2 as a function of the plasma irradiation time. The measurement was performed for three areas on every wafer. As is shown in this figure, in case of the oxygen plasma, the surface roughness is reduced in all wafers at an irradiation time of 10–30 s compared with the initial pre-cleaned state. In contrast, the surface roughness of Ce:YIG, InP and LiNbO_3_ wafers increases in case of the argon plasma. From these observations, it can be concluded that the oxygen plasma irradiation for 10–30 s is best for reducing the roughness of Ce:YIG, SOI, InP and LiNbO_3_ surfaces.

The oxygen ion bombardment makes the wafer surface smooth with a physical reaction. Also, it is reported that the oxidation process forms an active oxide layer on the wafer surfaces in the oxygen plasma irradiation [37]. This increases the surface energy of wafers, which contributes to the enhancement of wafer adhesion.

**Figure 3:**
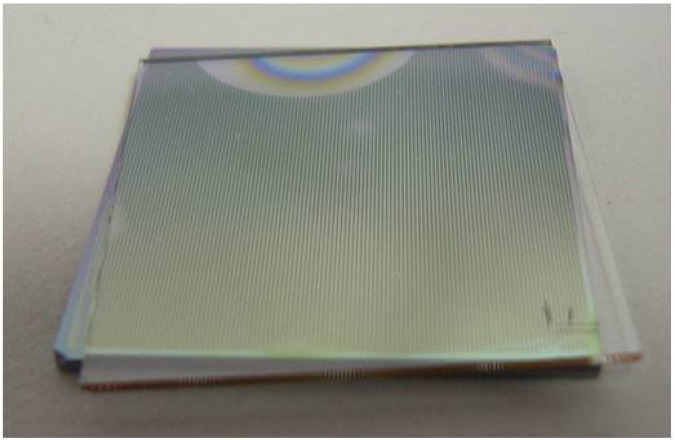
Ce:YIG on SOI sample fabricated by the surface activated direct bonding technique [38].

After the surface activation process of the oxygen plasma irradiation of 30 s and 10 s for Ce:YIG and SOI, respectively, the wafers were brought into contact in a vacuum chamber. The contacted wafers were pressed with a pressure of 5 MPa for 8 h. During this process, the sample was heated at a temperature of 200 °C on a Ce:YIG side and 250 °C on an SOI side. It should be noted that a relatively low temperature process is crucial for bonding dissimilar crystals in order to circumvent the problems associated with the mismatch in thermal expansion. The bond strength was measured to be <0.02 MPa by a tensile test for the bonded Ce:YIG/SOI sample with an activation process in the oxygen plasma generated with an RF power of 400 W. The bond strength was improved by exposing the wafers to the plasma generated with a higher RF power. The bond strength >1.8 MPa was obtained, when 500 W RF power was applied to generate the oxygen plasma. Such a high bond strength is attributable to the increase in surface activation energy. There was no significant degradation in the surface roughness of wafers after the plasma irradiation generated with an RF power of 500 W [36]. The bonded sample is shown in Figure 3.

In another series of experiments, a Ce:YIG wafer was bonded onto a GaInAsP (λ_g_ = 1.25 μm) layer grown on a (100) InP substrate. The thickness of the GaInAsP layer was 0.45 μm. 9 × 10 mm^2^ wafers were used in this study. After the surface activation process of oxygen plasma irradiation, two wafers were brought into contact, and were pressed with a pressure of 1.0 MPa at a temperature of 250 °C for 1 h. When a tensile >0.5 MPa was applied to the bonded sample, fracturing was observed in the InP substrate without debonding between Ce:YIG and GaInAsP [34]. It can be concluded that the bond strength is >0.5 MPa.

## 4. Interferometric Waveguide Isolator

As is described in Section 2, in the early stage of waveguide optical isolator research, a TE-TM mode conversion waveguide optical isolator was studied, which, in principle, is the same as the polarization rotation in a bulk Faraday isolator [13,14,15,16,17,18,19,20]. The phase matching should be achieved between TE and TM modes so as to obtain the mode conversion sufficient for isolator operation. A birefringence-free waveguide is realizable by balancing the various contributions of birefringence, e.g., geometrical, stress-induced, and growth-induced birefringence [19]. Although, in principle, it is possible by carefully controlling the various contributions of birefringence, the tolerances in the manufacturing process make it impractically difficult to achieve the required control. Also, the operation wavelength range is limited, since the phase matching condition is sensitive to wavelength.

In order to alleviate the phase matching issue, the interferometric waveguide optical isolator is adopted, in which the magneto-optic phase shift is used in a Mach-Zehnder interferometer to obtain the nonreciprocal transmission characteristic [39]. The interferometric isolator has the advantage of single polarization operation, which results in no need for the TE-TM mode phase matching. 

Mizumoto *et al*. measured the magneto-optic phase shift for TM mode propagating in magneto-optic garnet waveguides composed of YIG [40] and bismuth-substituted gadolinium iron garnet (Bi:GdIG) [41]. The performance of the interferometric waveguide isolator was demonstrated in the waveguide where a magneto-optic garnet was used as a guiding layer [42,43,44]. The magneto-optic phase shift is obtainable even in the waveguide where a magneto-optic material is loaded as a cladding layer [45]. The surface activated direct bonding technique was developed to integrate a magneto-optic garnet on SOI and III-V compound semiconductors as is described in the previous section. Using this technique, the interferometric waveguide isolator is realizable in an SOI waveguide as well as a III-V compound semiconductor waveguide. 

**Figure 4:**
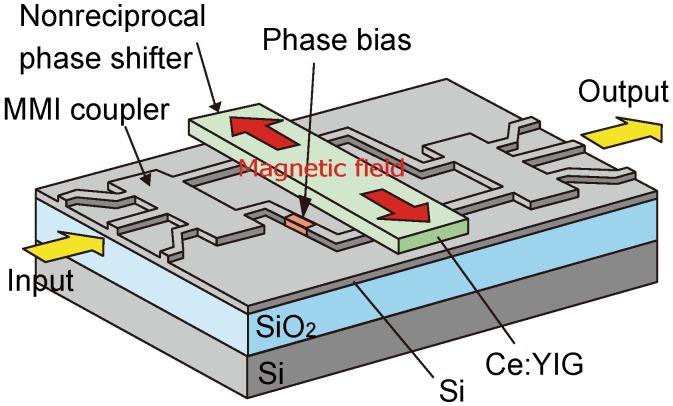
The schematic drawing of SOI interferometric waveguide optical isolator [46].

The schematic structure of a SOI interferometric waveguide isolator [46] is shown in Figure 4. A Mach-Zehnder interferometer is composed of multi-mode interference (MMI) couplers for dividing and combining light waves. Magneto-optic phase shifters, installed in the waveguide arms of interferometer, are constructed with a Ce:YIG cladding layer directly bonded on a silicon guiding layer. An external magnetic field is applied transversely to the light propagation direction so as to saturate the magnetization of Ce:YIG in-plane. Due to the magneto-optic effect originating from the in-plane magnetization, a change in the propagation constant of TM modes is induced while propagating in the waveguide. The change in the propagation constant can be obtained by reversing the propagation direction as well as the applied magnetic field direction. It should be noted that external magnetic fields are applied in anti-parallel directions in two arms of the interferometer. This results in different magneto-optic phase shifts in the two arms. That is, the light wave propagating from left to right experiences a phase shift of Φ_+_ and Φ_−_ in the upper and the lower arm, respectively. When the propagation direction is reversed, the light wave undergoes the phase shift of Φ_−_ and Φ_+_ in the upper and the lower arm, respectively. This can be understood by considering the relation between the propagation direction of the light wave and the direction of the magnetic field applied to each waveguide arm.

The phase difference is brought about between two interferometer arms by virtue of such a magneto-optic effect called the nonreciprocal phase shift. By properly choosing the length of nonreciprocal phase shifter, the phase difference, measured in the upper arm with respect to the lower arm, can be set to Φ_+_ − Φ_−_ = π/2 for the light wave propagating from left to right. This phase difference is cancelled by π/2 phase bias installed in the lower arm. The phase bias is generated by the optical path difference between the two arms, simply by adjusting the length and/or the width of the waveguide. As a result, the light wave propagating in two waveguide arms becomes in phase, and interferes constructively with the output MMI coupler located on the right. The light wave launched into the input port of the left MMI coupler emerges from the output port of the right MMI coupler. This corresponds to a forward direction.

In the backward direction, a phase difference given by the magneto-optic effect changes its sign. That is, the magneto-optic phase difference measured in the upper arm with respect to the lower arm becomes −π/2. As a result, the total phase difference between the two interferometer arms amounts to −π, since the phase bias remains given the −π/2 phase difference. Destructive interference occurs in the left MMI coupler. The light wave is not output from the initial input port, but is radiated out of the side of the waveguides.

The nonreciprocal phase shift Φ_+_− Φ_−_ is calculated by using (1), which is derived from the perturbation theory [47,48].

(1) $$\Phi_{+}-\Phi_{-}=\frac{\int \varepsilon_{0}\gamma \frac{\partial }{\partial x}\frac{{\left|H_{y}\right|}^{2}}{n^{4}}dS}{\int \frac{1}{n^{2}}{\left|H_{y}\right|}^{2}dS}$$

where *H_y_* denotes the distribution of the transverse magnetic field component of the TM mode guided in a waveguide. Gamma (γ) represents the off-diagonal component of the permittivity tensor of magneto-optic material, which is related to the specific Faraday rotation *θ*_F_ through (2).

(2) $$\gamma =\frac{n\lambda }{\pi }\theta_{F}$$

*n*, ε_0_ and λ are the refractive index of magneto-optic material, the permittivity of vacuum and the wavelength of light, respectively.

**Figure 5:**
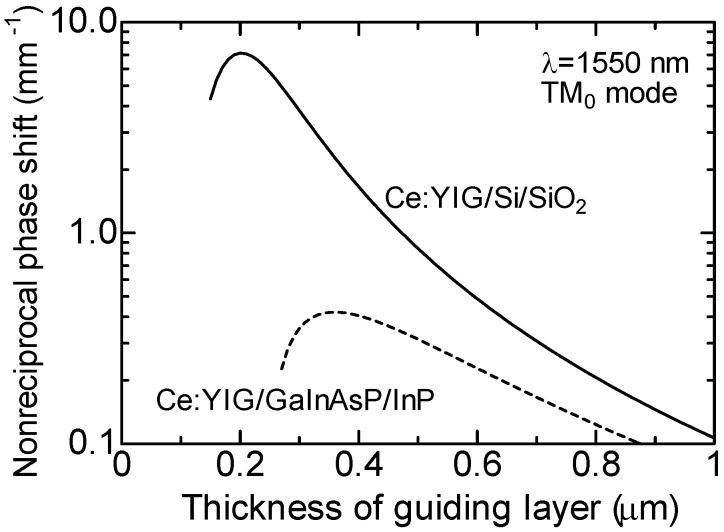
Calculated nonreciprocal phase shift of TM_0_ mode propagating in Ce:YIG/Si/SiO_2_ and Ce:YIG/GaInAsP/InP slab waveguides as a function of the thickness of guiding layer at a wavelength of 1,550 nm [38]. The specific Faraday rotation of Ce:YIG is assumed to be *θ*_F_ = −4,500°/cm.

The nonreciprocal phase shift is calculated in an SOI waveguide bonded with a Ce:YIG cladding layer. The slab waveguide is constructed with a layered structure of Ce:YIG/Si/SiO_2_. Here, the thickness of a buried oxide (SiO_2_) layer in the SOI wafer is assumed to be large enough so that a silicon substrate does not affect a fundamental guided mode. The refractive indices of Ce:YIG, Si and SiO_2_ are assumed to be 2.20, 3.48 and 1.44, respectively, at a calculated wavelength of 1550 nm. The specific Faraday rotation of Ce:YIG is assumed to be *θ*_F_ = −4,500°/cm [49]. Figure 5 shows the calculated nonreciprocal phase shift as a function of the thickness of silicon guiding layer. The nonreciprocal phase shift of a Ce:YIG/GaInAsP/InP slab waveguide is also shown in the figure for comparison. The refractive index of the GaInAsP guiding layer is assumed to be identical to that of silicon. It can be observed that the nonreciprocal phase shift takes a maximum value, when a waveguide is close to cutoff. It should be noted that an SOI waveguide provides a larger nonreciprocal phase shift compared with a GaInAsP/InP waveguide. This can be understood by the difference in the refractive index of the under cladding layer. That is, in case of the SOI waveguide, the refractive index of the under cladding layer SiO_2_ is 1.44, whereas it is 3.17 in case of the GaInAsP/InP waveguide. Since the refractive index of SiO_2_ is lower than that of Ce:YIG, the larger penetration of the optical field into a magneto-optic layer, *i.e.*, a larger magneto-optic effect, is obtainable in the SOI waveguide compared with the GaInAsP/InP waveguide [50,51]. In case of an SOI waveguide, a nonreciprocal phase shift of 7.12 mm^−1^ is obtainable for a 200-nm-thick silicon guiding layer at a wavelength of 1,550 nm.

In a practical device, a three-dimensional waveguide such as a rib or a rectangular core waveguide is used for the lateral optical field confinement. The nonreciprocal phase shift of the fundamental TM-like mode is calculated in a silicon rectangular waveguide, bonded with a Ce:YIG upper cladding layer, for various core widths in [38]. Compared with the slab waveguide, the nonreciprocal phase shift is decreased in three-dimensional waveguides, because the transverse field component in the Ce:YIG cladding layer is decreased. Although this tendency becomes more prominent for narrower waveguides, the difference from the slab waveguide is not remarkable in case of a 2-μm-wide rectangular silicon waveguide. In case of a silicon rib waveguide, the nonreciprocal phase shift takes a value between the slab and the rectangular waveguide of the same width. The length of nonreciprocal phase shifter providing Φ_+_− Φ_−_ = π/2 takes a minimum value of 220 μm in a 200-nm-thick silicon slab waveguide. When the thickness of the silicon guiding layer is 300 nm, the length of nonreciprocal phase shifter is 413 μm and 418 μm for a slab and a 2-μm-wide rectangular waveguide, respectively. A 2-μm-wide and 300-nm-thick rib waveguide with a rib height of 10 nm is used in the following fabrication. The nonreciprocal phase shift given in this waveguide is well approximated by that of a slab waveguide.

Although the nonreciprocal phase shift is provided only for TM modes in a vertically asymmetric waveguide, the polarization independent operation is realizable by adopting a proper design of an interferometric waveguide [52]. Also, the polarization independent operation is obtainable by introducing an asymmetric structure both in-plane and out-of-plane in a properly magnetized magneto-optic waveguide [53].

From a practical viewpoint, it is highly desirable for the isolator to operate in a wide wavelength range. The wavelength dependence of the interferometric waveguide isolator is mainly determined by the wavelength dependences of the nonreciprocal phase shift and the phase bias. The former is mainly governed by the wavelength dependence of the magneto-optic effect, which is reduced at longer wavelength regions. The latter is determined by the dispersion characteristic of waveguides. *Shoji et al.* [54] proposed to cancel two wavelength dependences by properly designing the interferometer. The wavelength dependence of the nonreciprocal phase shift can be canceled by that of phase bias in a wide wavelength range by introducing a phase bias of 3π/2 instead of π/2 and reversing the sign of nonreciprocal phase shift. The wideband operation was demonstrated in an interferometric waveguide isolator composed of a Ce:YIG guiding layer [55]. Also, by properly adjusting the waveguide widths and lengths of interferometer arms, an isolator can be realizable that covers both 1.31 and 1.55 μm wavelength ranges with an isolation >40dB in a single device [56]. 

An SOI waveguide optical isolator was fabricated in the SOI wafer that has a 300-nm-thick silicon layer and a 3-μm-thick buried oxide layer. Here, the thickness of the buried oxide layer was chosen so that a silicon substrate did not affect a fundamental TM mode propagating in the silicon layer. The silicon waveguide pattern was drawn using an electron beam lithography system. The waveguide mask was transferred into an evaporated Cr through a standard lift-off process. The silicon rib waveguide of 10 nm in height and 2 μm in width was formed in the 300-nm-thick silicon guiding layer using a reactive ion etching technique with a mixture gas of CHF_3_ and O_2_ (15:1).

A three-pole magnet system composed of a pair of compact permanent magnets was used in order to apply magnetic fields in anti-parallel directions in the two arms of the interferometer. A distance of 300 μm was chosen between the two arms of the interferometer for accommodating the magnet. Bending waveguides with a curvature radius of 0.5 mm were used, since the waveguide had a weak confinement of the optical field in the lateral direction. Because of this, the total device length was approximately 4 mm, though the required length of the nonreciprocal phase shifter was just 413 μm at a wavelength of 1,550 nm.

A 0.5-μm-thick Ce:YIG single-crystalline layer grown on a (111)-oriented SGGG substrate was directly bonded as an upper cladding layer using the surface activated direct bonding technique. After oxygen plasma was applied to the surfaces of Ce:YIG and silicon for 30 s as a surface activation process, the surfaces were contacted in a vacuum chamber. A pressure of 5 MPa was applied to the contacted sample gradually at a temperature of 250 °C for 1 h.

**Figure 6:**
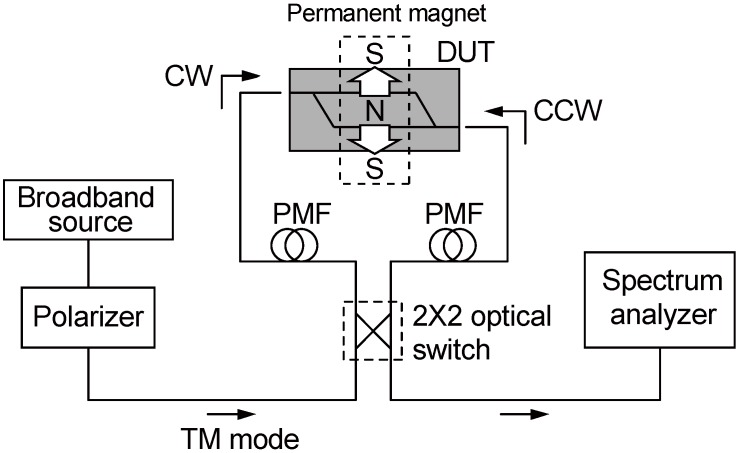
The measurement setup for the fiber-to-fiber transmittance of a waveguide optical isolator [46]. PMF: polarization maintaining fiber.

The fabricated isolator was characterized in the measurement setup shown in Figure 6. A fundamental TM mode emitted from a broadband light source was launched through a polarization maintaining fiber (PMF). The transmitted output from the device under test was probed by another PMF to measure its spectrum. The propagation direction was reversed by using a 2 × 2 optical switch to measure the forward and backward transmittance.

**Figure 7:**
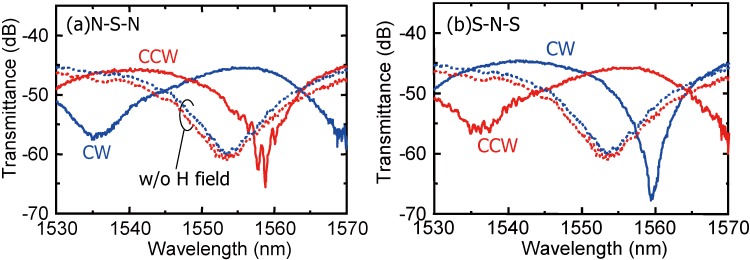
Measured fiber-to-fiber transmittance of a fabricated SOI interferometric waveguide optical isolator with the applied magnetic field by (**a**) N-S-N and (**b**) S-N-S three-pole permanent magnets [46]. Dotted lines show the transmittance without applying a magnetic field.

Figure 7 shows the measured fiber-to-fiber transmittance under a fixed applied magnetic field. The transmittance differs remarkably depending on the propagation direction. When the direction of magnetic field was reversed, the interference was also reversed [46]. This implies that the sign of magneto-optic phase shift was reversed by reversing the magnetic field direction. The maximum isolation defined by the transmittance ratio of the forward to the backward direction was measured to be 21 dB at a wavelength of 1,559 nm. Also, the insertion loss was 8 dB excluding 37 dB fiber-to-waveguide coupling loss at input and output interfaces.

## 5. Semileaky Waveguide Optical Isolator

A semileaky waveguide optical isolator was first proposed by S. Yamamoto *et al*. [57]. The isolator uses the semileaky waveguide structure, where TE and TM modes travel as guided and leaky modes, respectively. Leaky TM modes have continuous spectra of propagation constant. Thus, a guided TE mode can be phase matched to a leaky TM mode, even when dimensional deviations occur in a waveguide. That is, the fabrication tolerance is greatly relaxed to obtain a mode conversion between guided TE and leaky TM modes [58]. Also, a wide operation wavelength range is obtainable in the semileaky waveguide optical isolator. However, practically good performance has not been realized for a long time, because it was difficult to achieve a uniform and tight contact between a birefringent crystal and a magneto-optic garnet [59].

**Figure 8:**
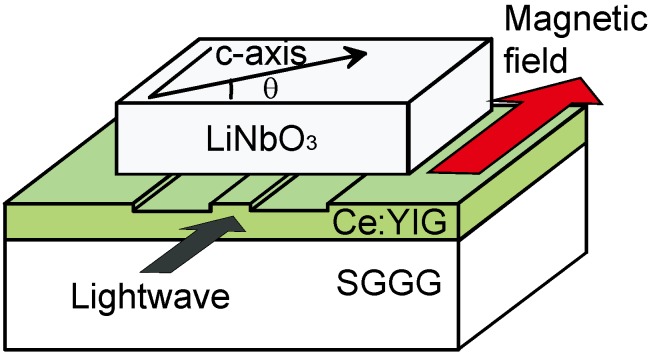
Schematic drawing of a semileaky waveguide optical isolator composed of a Ce:YIG guiding layer with a directly bonded LiNbO_3_ cladding layer [12].

A schematic drawing of a semileaky waveguide optical isolator is shown in Figure 8. The isolator consists of a magneto-optic garnet guiding layer and an upper cladding layer of a birefringent crystal such as LiNbO_3_. A Ce:YIG layer grown on a (111)-oriented SGGG substrate performs a guiding layer. The crystal axis of a LiNbO_3_ cladding layer is in the plane parallel to the interface of the guiding and cladding layers. When the offset angle of crystal axis *θ* is small, TE modes experience the extra-ordinary refractive index of LiNbO_3_ (*n*_e_ = 2.14) that is lower than the index of Ce:YIG guiding layer (*n* = 2.20). In contrast to this, TM modes are influenced by the ordinary index of LiNbO_3_ (*n*_0_ = 2.21) that is higher than the guiding layer’s index. Thus, TE modes are confined in the guiding layer, whereas TM modes are radiated into the LiNbO_3_ cladding layer.

When an external magnetic field is applied along the light propagation direction, a magneto-optic TE-TM mode conversion occurs, which corresponds to the Faraday rotation. Also, LiNbO_3_ gives a conversion between TE and TM modes. The magneto-optic mode conversion is nonreciprocal, *i.e.*, dependent on the propagation direction, whereas the mode conversion associated with LiNbO_3_ is reciprocal, *i.e.*, independent of the propagation direction. By properly choosing the offset angle of the LiNbO_3_ crystal axis, the magneto-optic mode conversion is canceled by the LiNbO_3_ mode conversion in the forward propagation. In the backward propagation, there remains the mode conversion because of the nonreciprocal nature of the magneto-optic mode conversion. The forward propagating TE mode is guided without being converted into leaky TM modes. On the other hand, the backward propagating TE mode is converted into TM modes and is attenuated due to the radiation into the LiNbO_3_ cladding layer. Therefore, the device works as an optical isolator for a TE mode.

**Figure 9:**
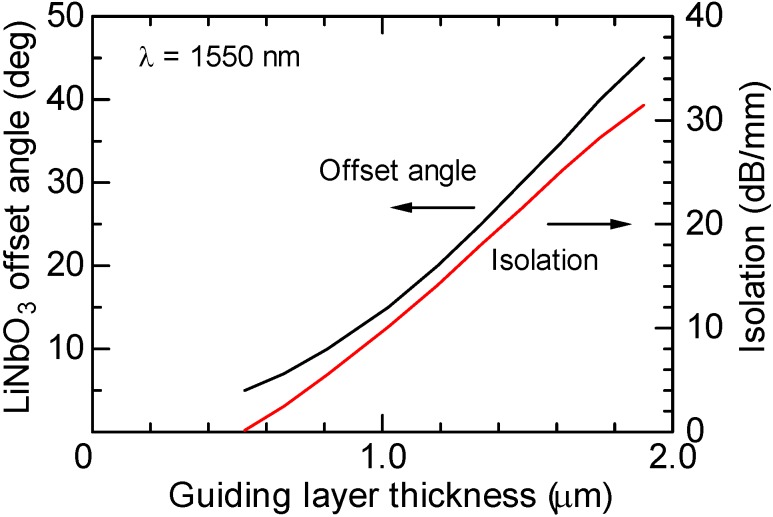
Calculated offset angle of LiNbO_3_ crystal axis which is determined to cancel the forward mode conversions as a function of the thickness of the Ce:YIG guiding layer at a wavelength of 1,550 nm [12]. Also, an obtainable optical isolation is plotted.

The offset angle of the LiNbO_3_ crystal axis is determined by the condition to cancel the forward mode conversion. Figure 9 shows the offset angle calculated at a wavelength of 1,550 nm as a function of the guiding layer thickness. The specific Faraday rotation of Ce:YIG is assumed to be *θ*_F_ = −4,500°/cm. When the thickness of the guiding layer is 1.19 μm, which supports only a fundamental TE guided mode, the offset angle is determined to be 20° for cancelling the forward mode conversion.

**Figure 10:**
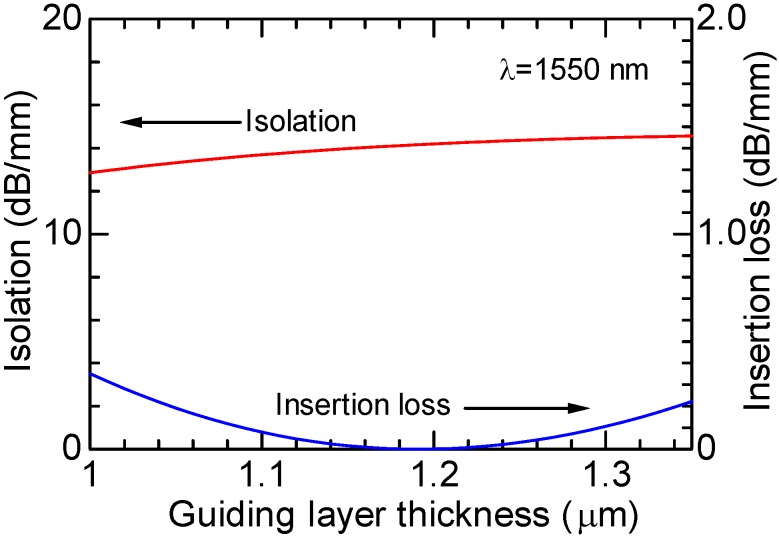
Calculated dependence of isolation and insertion loss on the thickness of Ce:YIG guiding layer of semileaky waveguide optical isolator at a wavelength of 1,550 nm [12].

The dependence of isolation on the deviation of guiding layer thickness is calculated at a wavelength of 1,550 nm as shown in Figure 10, where the offset angle of the LiNbO_3_ crystal axis is set to be 20° [12]. For a thickness deviation of +/−0.1 μm, the increase in an insertion loss is less than 0.1 dB/mm, and an isolation >13.6 dB/mm is obtainable. It can be said that the semileaky isolator has a large fabrication tolerance. Forward and backward losses increase in proportion to the propagation distance, since they are given by radiation losses.

**Figure 11:**
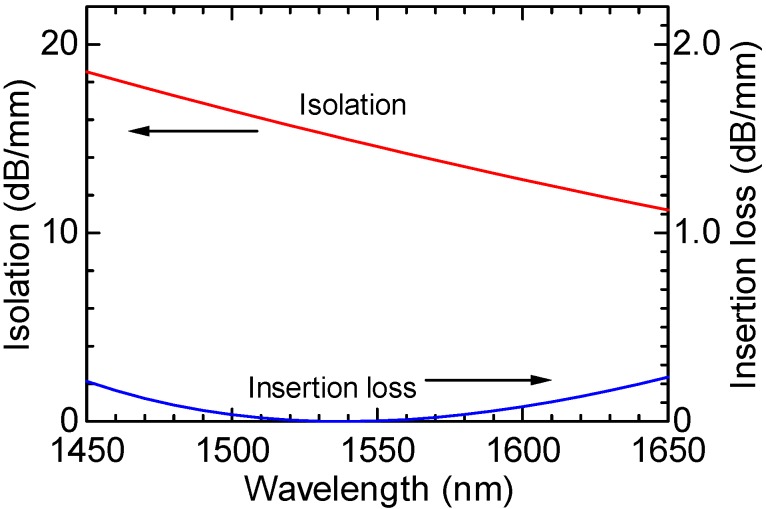
Calculated dependence of isolation and insertion loss on a wavelength in the semileaky waveguide optical isolator, where the thickness of the Ce:YIG guiding layer and the offset angle of the LiNbO_3_ crystal axis are set to be 1.19 μm and 20°, respectively [12].

The wavelength dependence of isolator performance is shown in Figure 11, where the thickness of the Ce:YIG guiding layer and the offset angle of the LiNbO_3_ crystal axis are set to be 1.19 μm and 20°, respectively [12]. In a shorter wavelength region, a larger backward loss is obtained due to an increased magneto-optic mode conversion. An isolation >12 dB/mm is obtainable together with an insertion loss <0.16 dB/mm in S-, C- and L-bands of optical fiber communications.

A semileaky waveguide optical isolator was fabricated with a 3-μm-wide Ce:YIG rib waveguide on which x-cut LiNbO_3_ was directly bonded as an upper cladding layer [12]. The thickness of Ce:YIG guiding layer was 1.19 μm, and the rib height was 0.1 μm. LiNbO_3_ was successfully bonded on Ce:YIG by using the surface activated bonding technique. In a surface activation process, Ce:YIG and LiNbO_3_ wafers were exposed to a plasma generated in a mixture gas of Ar and O_2_ for 5 min. An RF power of 250 W was applied to the 40 × 30 mm^2^ electrode on which two wafers were placed. Successful bonding was achieved by contacting activated wafer surfaces in a vacuum chamber and by applying a pressure of 1 MPa for 3 min at room temperature. The LiNbO_3_/Ce:YIG interaction length along the light propagation direction was 1.5 mm in a fabricated device.

**Figure 12:**
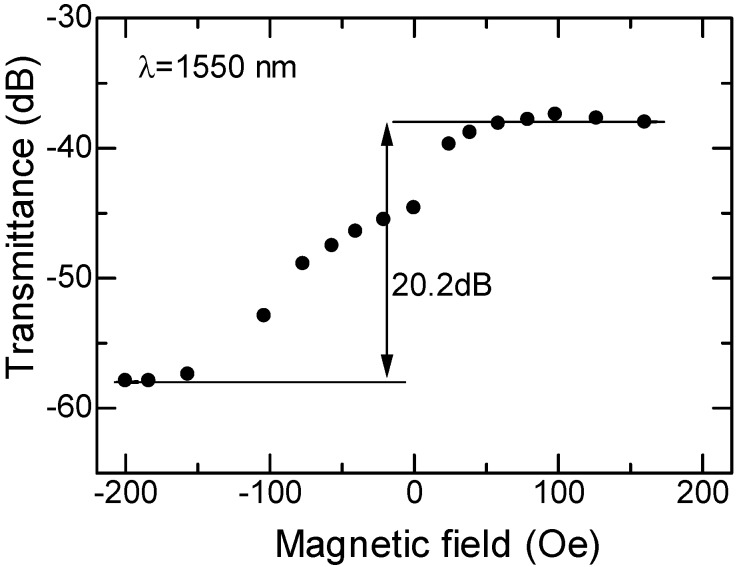
Measured fiber-to-fiber transmittance of semileaky waveguide optical isolator as a function of applied magnetic field at a wavelength of 1,550 nm [12]. A coupling loss of 15 dB/facet between the butt-coupled PMF and the waveguide is included in the transmittance.

The TE_0_ mode of 1,550 nm wavelength was launched in a device through a butt-coupled PMF. The transmitted power was probed with another PMF butt-coupled to the waveguide output facet. An external magnetic field was applied along the light propagation direction to generate the magneto-optic mode conversion. The measured fiber-to-fiber transmittance is shown in Figure 12 as a function of the applied magnetic field. It should be noted that a coupling loss of 15 dB/facet between the PMF and the waveguide is included in the measured transmittance.

Reversing the direction of the applied magnetic field is equivalent to the reversal of the light propagation direction. When the magnetic field is applied in the forward direction, the transmittance is increased. Contrary to this, it is decreased, when the direction of magnetic field is reversed. This is due to the fact that the mode conversion induced by the magneto-optic effect cancels the conversion associated with LiNbO_3_ in the forward direction and is added to the LiNbO_3_ conversion in the backward direction. An isolation of 20.2 dB, which corresponds to 13.5 dB/mm, was obtained by comparing the transmittance between opposite directions with an applied magnetic field of +/−200 Oe. The measured isolation of 13.5 dB/mm is comparable to the calculated value of 14.1 dB/mm.

## 6. Conclusions

In this paper, we have reviewed the direct bonding technique for the waveguide optical isolator application. The surface activated direct bonding technique is developed for integrating a magneto-optic garnet onto commonly used waveguide platforms of SOI and III-V compound semiconductor wafers. This technique has the potential advantage that dissimilar materials are bonded at low temperatures enabling the avoidance of the issue associated with the difference in thermal expansion. It was shown that oxygen plasma irradiation was effective as a surface activation process for bonding a magneto-optic garnet on SOI and III-V compound semiconductor wafers. Using this technique, the interferometric waveguide optical isolator and the semileaky waveguide optical isolator were fabricated with good performance characteristics. It can be concluded that the direct bonding technique is versatile for realizing waveguide optical isolators in which a magneto-optic garnet is used to obtain the nonreciprocal function.

The use of bonding has the advantage that materials can be chosen to be optimized for the performance of devices. However, the preparation of a flat region of a substantially large area is needed for stable bonding, which is undesirable for the simplicity of fabrication process. There is no such requirement in the case of deposition. Developing the deposition of a magneto-optic material with low optical absorption and large magneto-optic effect will contribute greatly to further progress in integrating optical isolators. Alternatively, a non-magneto-optic configuration is another approach to overcome the material constraints [60]. 

As for the performance characteristics of waveguide optical isolators, further studies must be done for the realization of polarization independent operation. Also, reducing the device size is important from a practical viewpoint. The use of magnetophotonic crystals is an interesting approach, though it is still in the theoretical stage [61,62].
